# mHealth-Based Gamification Interventions to Promote Health Among Older Adults: Scoping Review

**DOI:** 10.2196/82368

**Published:** 2026-05-04

**Authors:** Xi Chen, Yishu Zhu, Dian Jiang, Jundan Huang, Qi Xie, Yishi Chen, Jing Chang, Weiping Huang, Hongting Ning, Hui Feng

**Affiliations:** 1 Xiangya School of Nursing, Central South University, No. 172, Tongzipo Road, Yuelu District, Changsha, 410013, China, 86 15173121969; 2 Teaching and Research Section of Clinical Nursing, Xiangya Hospital of Central South University, Changsha, China; 3 School of Nursing, Hunan University of Chinese Medicine, Changsha, China

**Keywords:** mHealth, gamification, health promotion, older adults, health behavior, mobile health

## Abstract

**Background:**

Healthy aging has emerged as a global priority. However, older adults’ participation in health promotion programs remains low, and traditional health promotion models have achieved limited success in fostering sustained engagement among this population. Mobile health (mHealth)–based gamification interventions offer a promising way to address these challenges. However, no published reviews support or oppose the use of mHealth-based gamification interventions as health promotion strategies in older adults.

**Objective:**

The study aimed to identify mHealth interventions using gamification to promote health among older adults.

**Methods:**

Our scoping review was conducted following the Joanna Briggs Institute recommendations for scoping reviews and Arksey and O’Malley’s framework. The process followed PRISMA-ScR (Preferred Reporting Items for Systematic Reviews and Meta-Analyses Extension for Scoping Reviews) guidelines and PRISMA-S (Preferred Reporting Items for Systematic Reviews and Meta-Analyses Literature Search Extension) checklist. A comprehensive literature search was conducted across 8 databases: PubMed, Scopus, Web of Science, Embase, Cochrane Library, CINAHL, PsycARTICLES, and IEEE Xplore Digital Library, from their inception to December 10, 2025. Two reviewers independently screened titles, abstracts, and full texts via Rayyan, with disagreements resolved by a third reviewer.

**Results:**

This scoping review identified 11 studies. Only 1 article was published before 2022. The interventions were found to improve enjoyment and motivation (n=5), cognitive function (n=3), physical activity (n=2), and digital literacy (n=2). Individual studies also reported improvements in mental health (n=1) and adherence (n=1), a reduction in suicidal ideation (n=1), improvements in physical function (n=1), the promotion of social engagement (n=1), and the identification of mild cognitive impairment (n=1). Game elements used were ranked by frequency as progress, challenges, goals, levels, reward, sensation, storytelling or narration, leaderboard, surprise, and avatar. No research was found to use the game element of “social sharing.” mHealth types included augmented and virtual reality–based training systems, wearable devices, mobile phones, tablets, and Windows platforms and devices. Notably, only 4 studies applied theoretical frameworks, and 3 omitted the concrete approach to gamification.

**Conclusions:**

As the first scoping review to identify and map mHealth-based gamification interventions for older adults, this study highlights their potential as an innovative approach to health promotion. By systematically synthesizing evidence regarding intervention designs, gamification strategies, and preliminary health outcomes, it establishes a foundation for future inquiry. However, this review is limited by the small number of included studies, precluding broad generalizations. Future research should assess long-term impacts, integrate theoretical frameworks, establish reporting guidelines, design personalized social-interactive interventions, and expand to broader health domains. Ultimately, these insights provide targeted guidance for developing age-appropriate digital health solutions, contributing to the realization of active aging.

## Introduction

Globally, the proportion of older people continues to rise. By 2050, the number of people over the age of 65 years is expected to reach approximately one-sixth of the total population [1]. The aging process often leads to cognitive and physical changes in older adults that, if not addressed, can lead to a variety of health complications, including dementia [2]. Maintaining the health of older people is not only related to the quality of individual life but also the core issue of coping with the social and economic pressure of aging.

Healthy aging has also become a global priority, with the World Health Organization identifying the promotion of older people’s health as one of its sustainable development goals [3]. However, the number of older people participating in health promotion programs is small [4,5]. In addition, traditional health promotion models have had limited success in engaging older persons in sustained engagement. Therefore, there is an urgent need for innovative interventions to improve the health behaviors of older persons.

Mobile health (mHealth) is showing its unique potential as an effective way to address these challenges. As a new and rapidly growing field, mHealth enables the implementation of predefined health promotion interventions and practices through mobile devices and their applications [6]. mHealth is an ideal choice for health promotion because of its significant benefits: continuous availability of services, wider access, equity in access to services, personalization of content, lower cost, and increased service capability and efficiency [7]. In recent years, with the increasing penetration of smartphones and the overall increase in digital literacy among the public, public and private health care systems have made significant progress in using mobile apps to address public health challenges [7]. In this context, mHealth is an innovative tool that can greatly broaden access to health promotion services for older people and flexibly adapt to a wide range of health-related needs and contexts.

There is a growing interest in integrating game-like elements into mobile apps, known as gamification, to increase user engagement and enable personalization [8,9]. This review uses a definition provided by Deterding et al [10-12]. They define gamification as the use of game elements (such as goal-setting, progress bars, rewards, feedback, badges, points, competition, cooperation, leaderboards, etc) rather than mature games in nonplayable services and applications to enhance user experience and engagement [10-12]. While game elements are not always clearly distinguishable, they refer to the typical features of a game [10]. To be clear, this review focuses on gamification, not serious games, because serious games provide a pure gaming experience by creating complete and immersive games. In contrast to serious games, this article focuses not on the full game but on the fun elements.

Systems that incorporate gamification often use incentives to promote the adoption of mHealth technologies and healthy behaviors, including immediate success feedback (rewards), continuous progress feedback, and goal setting. For example, users receive badges as rewards when they win, and they can share their achievements on social networks [13]. In rehabilitation and behavioral sciences, gamification has been integrated into mHealth, virtual reality (VR) technology, and robotics and has been effective in motivating patients with cardiovascular disease [14-16], diabetes [17,18], and stroke [19-21] to participate more actively in long-term health management. Multiple studies have shown that mobile app–based gamification interventions can improve patients’ adherence to medical protocols and effectively manage their health conditions [22-24]. In addition, a literature review of empirical studies on gamification further confirms the effectiveness of gamification in improving user motivation and engagement [25], which is the core goal of many established behavior change strategies [26-30]. Taken together, gamification seems to be an effective strategy for promoting health [31].

Older people are becoming a growing segment of technology users with unique needs and challenges [32]. Furthermore, in the field of health care, there is a lack of understanding of gamification as a design strategy, and its application is often restricted to the use of video games [33] or blindly incorporating game elements without discrimination to motivate certain behaviors (such as physical activity) [34]. In addition, the concept of gamification is relatively new [10], and research on mHealth-based gamification interventions for the age group 60 years and older is still in the early stages of exploration. Therefore, for older people, more in-depth investigations and customized reviews are necessary. Unfortunately, in existing literature databases, we were unable to search for an English-language scoping review that explored the current use of mHealth-based gamification interventions to promote health in older adults.

To address these gaps, this study adopted a scoping review methodology to identify mHealth interventions using gamification to promote health among older adults. To provide a comprehensive overview, this review specifically analyzes (1) intervention characteristics and the health promotion topics addressed; (2) gamification characteristics; (3) the needs and experiences of older adults regarding these interventions; and (4) current knowledge gaps to provide recommendations for future research.

## Methods

### Overview

This study was structured according to the framework proposed by Arksey and O’Malley [35], with our methodology being guided by the steps described in the Joanna Briggs Institute manual for key phases, including study selection; data mapping; and the collation, synthesis, and reporting of results [36]. The reporting of this scoping review aligned with the PRISMA-ScR (Preferred Reporting Items for Systematic Reviews and Meta-Analyses Extension for Scoping Reviews) checklist (Checklist 1) and PRISMA-S (Preferred Reporting Items for Systematic Reviews and Meta-Analyses Literature Search Extension) checklist (Checklist 2) [37]. To ensure methodological rigor, inclusion and exclusion criteria were predefined prior to initiating the review process.

### Search Strategy

All authors discussed and decided to include the following 8 databases: PubMed, Scopus, Web of Science, Embase, Cochrane Library, CINAHL, PsycARTICLES, and IEEE Xplore Digital Library. The IEEE Xplore Digital Library is a more technology-oriented database that allows us to search for articles in the fields of informatics and engineering. A comprehensive literature search was conducted across all databases, covering the period from their inception to an initial search date of February 18, 2025. This search was subsequently updated by rerunning the same strategy on December 10, 2025, to identify any newly published relevant studies. The language of publication was limited to English to ensure an accurate understanding of the research.

The search strategy included terms across three categories: (1) mHealth (eg, telemedicine OR telehealth OR e-health OR m-Health OR eHealth OR mHealth OR “mobile applications” OR applications OR application OR app OR apps OR online OR mobile OR internet OR “web based” OR Smartphone OR “phone, smart” OR “smart phones” OR smartphones OR “smart phone” OR “phones, smart” OR “cell phone” OR iphone OR android OR iOS OR “Wearable Electronic Devices” OR website OR digital* OR system* OR electronic* OR technolog* OR device OR framework* OR “social support” OR Facebook OR “networks, social”), (2) gamification (eg, Gamification OR “game-based learning” OR gaming OR gamif* OR “game element*” OR game* OR “game mechanic*” OR gameful* OR “game design element*” OR “game-design element*” OR “game interface element*” OR “game feature” OR “game-like element*” OR “videogame element*”), and (3) older adults (eg, “older adult*” OR “older people” OR elderly OR “older population” OR “senior citizen*”).

The search terms were derived from Medical Subject Headings (MeSH) and free-text words. These keywords were then combined using Boolean operators (AND, OR) to ensure a comprehensive search. All search algorithms were modified and refined for each database. For a detailed search strategy, see Multimedia Appendix 1. A supplementary manual search was conducted by reviewing the reference lists of all included articles. Additionally, ResearchGate was used as a targeted supplementary tool for two specific purposes: (1) to retrieve the full texts of potentially eligible peer-reviewed articles that were otherwise inaccessible, including directly requesting full texts from the authors; and (2) to review the publication profiles of key authors from the included studies to identify any other relevant peer-reviewed journal publications meeting our inclusion criteria. The inclusion and exclusion criteria are mentioned in Textbox 1.

Textbox 1. **Inclusion and exclusion criteria.**

Inclusion criteria

- This review primarily includes interventions based on “gamification” rather than “serious games,” both of which are defined in the introduction. We explicitly did not exclude studies simply due to the absence of a clear definition of “gamification” or “serious games” within the text. Instead, we require that gamification be explicitly stated or at least 1 game element used in the article
- Peer-reviewed English-language journal publications with no date limit until the final search date
- Original empirical research, including qualitative and quantitative research (must be experimental research)
- Gamification is delivered through mHealth devices such as computers, smartphones, and wearables
- The purpose of gamification is to promote health in older adults, including but not limited to physical activity, cognitive function, mental health, nutritional intake, and social interaction
- The mean age of the participants included in the article is 60 years or older

Exclusion criteria

- Excluding reviews, protocols, design documents, letters, magazines, dissertations, abstracts, reviews, posters, preprints, conference proceedings, conference abstracts, nonprimary data sources, books, and book chapters
- Excludes serious or mature games (eg, video games, immersive virtual reality games, and augmented reality exergames)
- If the entire article cannot be accessed or retrieved, the study will also be excluded
- Exclude studies that only mention gamification superficially, for example, as a buzzword or as a suggestion for future research, without it being a core component of the study’s intervention or analysis

### Study Selection

We first used the software EndNote X21 (Clarivate) to automatically identify and remove duplicates. Then, we imported the deduplicated documents from EndNote X21 into Rayyan software and continued to perform manual deduplication in Rayyan software. Prior to the formal review, a calibration exercise was conducted on a random sample of one-fifth of the titles and abstracts. Two reviewers (XC and YZ) independently screened this sample to ensure consistency. Following a precedent set in similar reviews, an interrater agreement of nine-tenths was achieved before proceeding with the screening of the remaining articles [38]. The complete screening was performed independently by the 2 reviewers using Rayyan software. Initially, the 2 authors (XC and YZ) screened all study titles and abstracts for eligibility based on inclusion and exclusion criteria. Next, the 2 authors (XC and YZ) examined all full-text articles to determine whether they included the use of mHealth-based gamification interventions to promote health in older adults. Finally, a supplementary manual search was performed by looking at the list of references for the included articles and using ResearchGate. To ensure the uniformity of the screening, the authors conducted several training sessions in Rayyan with the co-reviewers. After each stage, the 2 authors (XC and YZ) cross-checked their results and discussed any differences. Any disagreements were resolved by a third researcher (HF).

### Data Extraction

Two researchers (XC and YZ) independently used the data extraction table of Excel and extracted and encoded study characteristics (first author name and year of publication, country, study design, and study objectives), participant characteristics (mean age, percentage of female participants, and target population), intervention characteristics (sample size, study setting, type of mHealth used, the theory used, and duration), gamification characteristics (definition of gamification, gamification elements, reasons for using gamification elements, and the concrete approach to gamification), and the needs and experiences of older adults with mHealth-based gamification interventions, key measurements, and study results. We extracted game elements based on the classification in the research by Aschentrup et al [31], including reward, sensation, progress, challenges, surprise, storytelling or narration, social sharing, level, leaderboard, goals, and avatar.

The extracted data were cross-checked between authors (XC and YZ), and any discrepancies within data were discussed. Differences were resolved through consultation with the third author (HF). The first author (XC) synthesized the extracted data for analysis. All data were presented using the data charts suggested by Arksey and O’Malley [35].

## Results

### Study Selection

**Figure 1.**
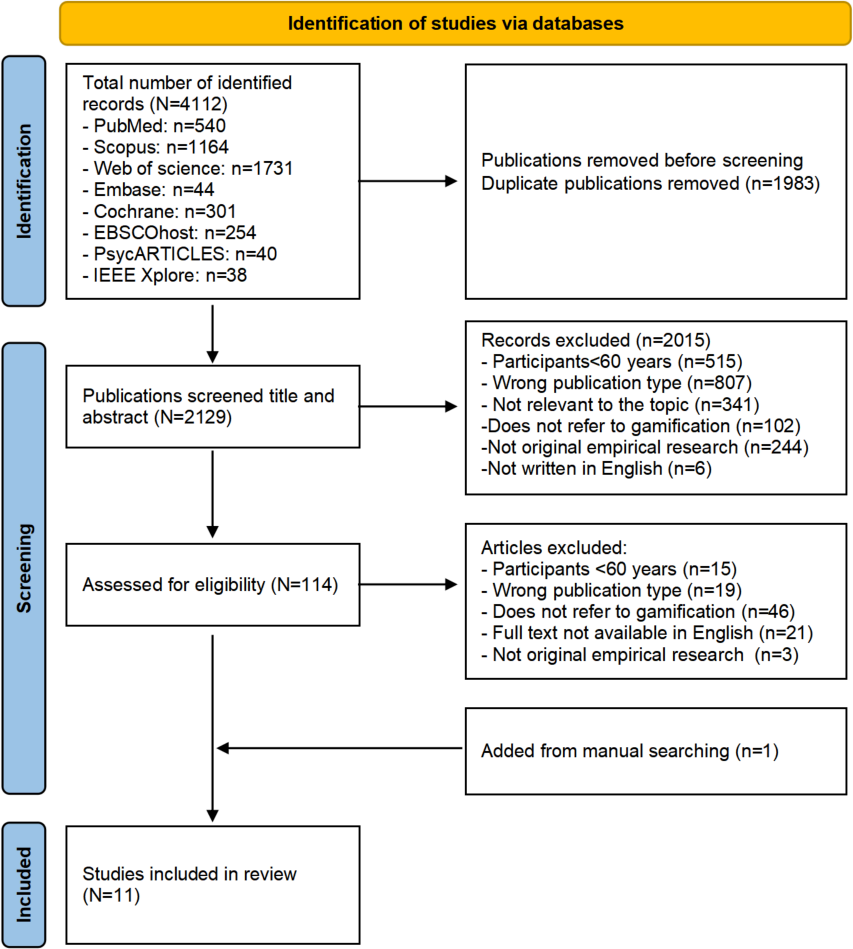
PRISMA (Preferred Reporting Items for Systematic Reviews and Meta-Analyses) flow diagram of article selection.

A total of 4112 records were found in our database. We first used the software EndNote X21 to automatically identify and delete duplicates, with 1928 duplicates removed. We then imported the deduplicated documents from EndNote X21 into Rayyan software, and continued to perform manual deduplication, which excluded 55 out of 2184 publications. The authors (XC and YZ) then used Rayyan software to screen all study titles and abstracts of these publications, achieving an interrater agreement rate of 0.978. The screening of titles and abstracts resulted in the exclusion of 2015 studies. The full texts of the remaining articles were then assessed for eligibility, resulting in the inclusion of 10 studies. An additional study was identified through a manual search of reference lists and using ResearchGate, bringing the final number of included studies to 11. The PRISMA (Preferred Reporting Items for Systematic Reviews and Meta-Analyses) flow diagram (Figure 1) provides more details on the screening process and reasons for exclusion.

### Study Characteristics

Table S1 in Multimedia Appendix 1 summarizes the research characteristics of the 11 articles included in this study. These studies were published between 2015 and 2025, with only 1 out of 8 articles published before 2022. These studies were distributed in the following 7 countries: China (3/11), South Korea (2/11), Switzerland (1/11), Spain (1/11), the United States (1/11), Japan (1/11), Thailand (1/11), and Malaysia (1/11). The research design included the following 3 types: 4 randomized controlled trials (RCTs), 4 quasi-experiments, 2 mixed methods studies, and 1 qualitative study. The research objectives of the included studies focused on developing mHealth-based gamification interventions for the older population and exploring their acceptability, feasibility, usability, safety, and effectiveness among older adults.

### Participant Characteristics

All included studies provided detailed reports on the female proportion, which was relatively high, ranging from 0.476 to 1.000. The 11 included studies recruited diverse populations of older adults. Three studies focused on participants with specific cognitive conditions, including those with early symptoms of memory disorders [39], individuals at risk for Alzheimer disease and related dementias [40], and older adults with no diagnosed cognitive impairments [41]. Another 2 studies targeted participants with specific physical or mental health statuses: high-risk, postdischarge patients with chronic pulmonary disease [42], and older women experiencing suicidal ideation [43]. A further study recruited hospital inpatients without specifying their condition [44]. The remaining 5 studies did not explicitly specify the health status of their participants.

### Intervention Characteristics

A total of 545 participants were included in this review, with sample sizes ranging from 12 to 138. The study setting covered communities (n=3), hospitals (n=3), homes (n=2), care centers (n=1), and universities (n=1). One study did not explicitly specify the study setting. The mHealth types comprised augmented and VR-based training systems, wearable devices, mobile phones, tablets, and Windows platforms and devices. Some studies used a single device, while others used a combination of technologies. Furthermore, only 4 studies used theory. These included the application of behavioral economic principles [40], gamification theory [45], technology acceptance model [41], and human-centered design framework [42]. Among all the studies, only 4 studies provided the duration of a single intervention, ranging from 5 minutes to 60 minutes. Three studies provided the intervention frequency, all of which were 3 times a week. Seven studies provided the total duration of the intervention, ranging from 3 to 14 weeks. For detailed information, please refer to Table S1 in Multimedia Appendix 1.

### Gamification Characteristics

**Figure 2.**
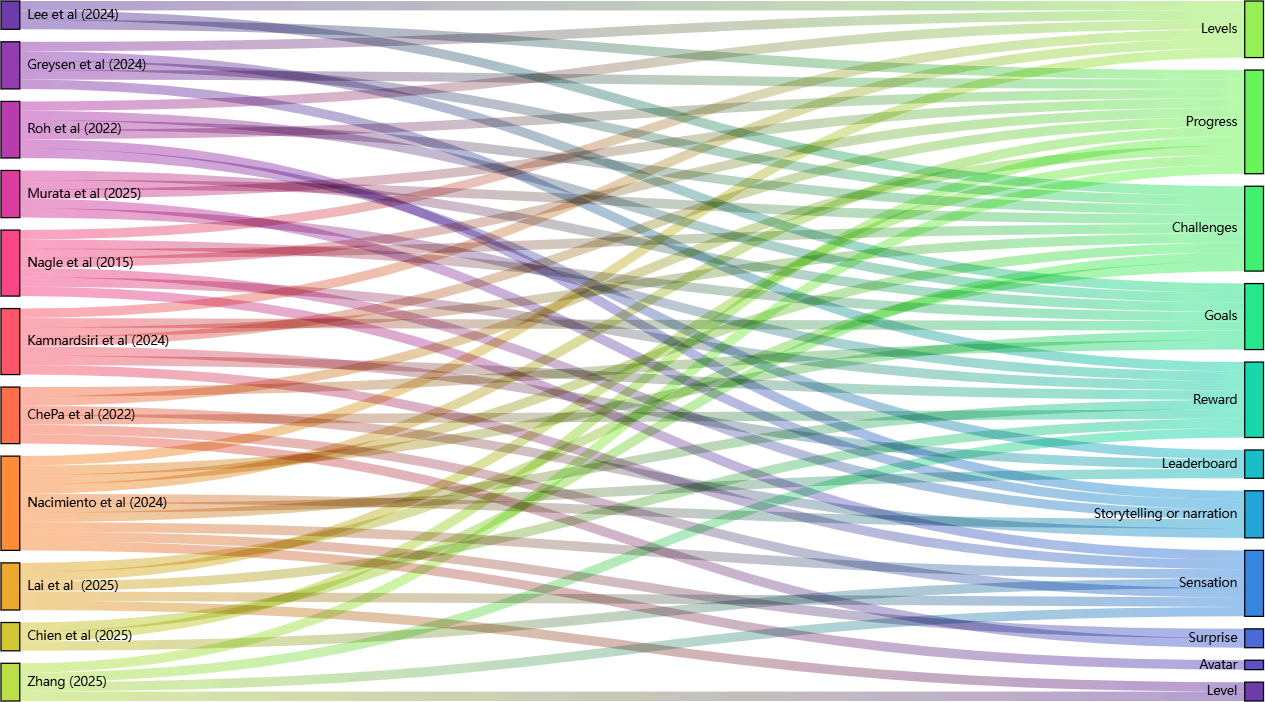
Sankey diagram illustrating the distribution and flow of game elements across the included studies. The left column represents the individual studies, while the right column lists the identified game elements. Each colored flow connects a study to the specific game elements it used [2,39-48].

Of the 11 included studies, only 5 provided a definition of gamification and were consistent with this review. The number of game elements used in these studies ranged from 1 to 11. Multimedia Appendix 2 illustrates the frequency of each game element across the included studies. Interestingly, no research was found to use the game element of “social sharing.” Figure 2 presents a Sankey diagram illustrating the specific many-to-many relationships between each study and the elements it used. Overall, each included study integrated at least 3 game elements, with the study by Nacimiento-García et al [2] using the highest number of game elements (n=10). The primary reasons cited for incorporating game elements were to promote engagement and motivation (n=7), enhance adherence (n=3), and increase enjoyment (n=3). Other reasons for using game elements are detailed in Table S2 in Multimedia Appendix 1. Additionally, 7 studies described “the concrete approach to gamification,” indicating that reporting on this aspect requires strengthening in future research.

### Needs and Experiences

Three included studies reported on user needs and experiences through qualitative methods. Positive user experiences, reported across the studies, included high acceptability, satisfaction, emotional satisfaction, enhanced enjoyment, motivation to continue use, a refreshing experience, perceived cognitive benefits, and improvements in physical activity, memory, and sleep quality, alongside practical design aspects such as clear instructions, good design, suitability for personal preferences, and accessibility for play at any time [2,48]. The interventions’ social and collaborative aspects were also described as valuable for fostering a sense of community [2].

Reported challenges and negative experiences included technical issues such as system freezes, difficulty with game mechanics, relatively small character and object sizes, and misalignment between in-game and actual movements, as well as experiences of stress and initial physical fatigue [2,48]. Furthermore, user needs and preferences were found to differ by gender, with men valuing cognitive challenges, while women emphasized practical memory support [41].

### Health Promotion Domains and Recommendations

#### Overview

The 11 included studies reported several positive outcomes. The interventions were found to improve enjoyment and motivation (n=5), cognitive function (n=3), physical activity (n=2), and digital literacy (n=2). Individual studies also reported improvements in mental health (n=1) and adherence (n=1), a reduction in suicidal ideation (n=1), improvements in physical function (n=1), the promotion of social engagement (n=1), and the identification of mild cognitive impairment (n=1).

#### Improve Physical Function

One RCT by Lee et al [46] compared the efficacy of an 8-week mHealth-based gamification intervention against conventional exercise in 40 community-dwelling older adults (aged 60‐85 years). The study found significant improvements in physical function in both groups, concluding that the gamified intervention produced comparable exercise effects to conventional exercise. Notably, the study examined mHealth-based gamification interventions as an integrated whole rather than evaluating individual game elements. Future research is needed to identify specific game elements most effective in enhancing older adults’ physical function.

#### Improve Adherence

In a 3-week home-based trial with 21 healthy older adults, Nagler et al [47] investigated the impact of user control over game design elements (difficulty adjustment, reward selection, and visual theme customization) on adherence to a memory training game. The study compared a “user-control” mode to an “automatic” mode and found that the user-control group demonstrated significantly higher play frequency, total engagement duration, and memory task accuracy. These findings suggest that user-driven gamification enhances adherence and performance. However, the study combined 3 game elements, preventing identification of individual effects, and its small sample of healthy, motivated participants limited generalizability, while the nonrandomized design hindered causal inference. Additionally, the research did not specify the concrete approach to gamification, leaving unclear how exactly game mechanics were integrated into the mHealth intervention.

#### Promote Physical Activity

Greysen et al [40] conducted a 12-week RCT with 94 older adults at risk for Alzheimer’s disease. They found that an mHealth-based gamification intervention with a support partner led to a significant increase in physical activity (1699 steps/day; *P*<.001) compared to controls (step goals with daily feedback), a gain that was partially sustained at a 3-week follow-up. However, the authors noted several limitations, including the short intervention and follow-up periods and the exclusion of nine participants post randomization, which could introduce bias.

The RCT by Roh et al [45] targeted older adults to evaluate a gamification-based smartphone app for promoting brain health. Over 8 weeks, 40 older adults were randomized to the app intervention or control group, with outcomes measured via physical activity questionnaires, cognitive scores, and the Situational Motivation Scale. The intervention group showed significant improvements in moderate-intensity physical activity (*P*=.01) and an 8-week physical activity difference (*P*=.04) versus controls. However, the study faced challenges, including a small sample size and a short intervention period, which may limit generalizability to long-term behavior change in older adults.

#### Identify Mild Cognitive Impairment

Murata et al [44] developed and tested a gamified mobile app, based on the N-back task, to screen for mild cognitive impairment in 138 older adults. The study found significant correlations between gameplay performance metrics (eg, swipe time, accuracy) and cognitive function (Mini-Mental State Examination scores). The authors concluded that their gamified app could identify mild cognitive impairment with an accuracy comparable to standard dementia screening tests. However, the study did not address participants’ past smart device experience, which may confound motor-based metrics. Additionally, reliance on device-specific factors (eg, swipe time) raises cross-device reproducibility concerns.

#### Improve Cognitive Function and Promote Digital Literacy and Social Engagement

Nacimiento-García et al [2] developed a tailored mHealth-based gamification intervention, with games targeting memory, attention, and problem-solving. A pilot study found the intervention effectively promoted cognitive function, digital literacy, and social engagement. However, the authors noted that advanced tools like VR glasses posed initial usability barriers for some older users, and they recommended that future research should expand participant diversity and conduct longitudinal controlled studies to assess long-term impacts.

ChePa et al [39] used an mHealth-based gamification intervention for older individuals with memory disorders and found that it led to significant cognitive improvement, enhanced memory recall, and improved digital literacy. A key limitation noted was the lack of a specific description of how the gamification was implemented.

In a qualitative study by Lai et al [41], participants reported perceived cognitive benefits from using a Cantonese-language gamified mobile app. However, the reported cognitive benefits were based on self-reported experiences only; no objective cognitive or usage data were collected, and the small sample size limits the generalizability of findings across different demographic subgroups.

#### Improve Enjoyment and Motivation

Nacimiento-García et al [2] found that gamified experiences were enjoyable in mHealth-based interventions. Roh et al [45] demonstrated that such interventions significantly enhanced intrinsic motivation (*P*=.01), which subsequently promoted physical activity (*P*=.01). Kamnardsiri et al [48] reported comparable benefits in a home-based, low-intensity, gamification-based interactive physical-cognitive training pilot; participants showed a significant increase in enjoyment (*P*=.001) over 4 weeks. However, this study focused on prototype usability and did not evaluate clinical physical or cognitive outcomes, nor did it specify the concrete approach to gamification.

Lai et al [41] reported that participants experienced emotional satisfaction and expressed motivation to continue using a gamified application; however, the study did not assess enjoyment or motivation using quantitative measures. Given that the study was exploratory and based on voluntary participation, potential self-selection bias should be taken into account when interpreting the results.

Chien et al [42] reported that participants in a home-based pulmonary telerehabilitation program using the Intelligent Pulmonary Rehabilitation Exercise System experienced increased engagement, motivation, and enjoyment through personalized feedback, collaborative goal-setting, and gamified exercise modules. However, the study used a single-group design with a modest sample size, evaluations were primarily conducted during hospitalization, and the absence of a control group or comparison with other treatment modalities limits generalizability.

#### Improve Mental Health and Reduce Suicidal Ideation

Zhang [43] conducted an RCT involving 120 older women with suicidal ideation to evaluate a gamified group-based VR physical activity intervention. The study concluded that this approach serves as a promising strategy to enhance psychological well-being and mitigate suicide risk. However, the intervention lacked a guiding theoretical framework. Critically, the study design did not allow for the identification of the specific effects of individual game elements. Furthermore, the reliance on indirect indicators rather than direct suicide assessment, combined with the absence of blinding and longitudinal follow-up, presents methodological limitations.

## Discussion

### Summary of Key Findings

This is the first scoping review to examine mHealth-based gamification interventions for health promotion among older adults. This scoping review identified 11 studies. We provide a comprehensive overview of mHealth-based gamification interventions for older people’s health promotion in countries like China, Korea, and the United States. Only 1 article was published before 2022. These studies demonstrated the interventions’ effectiveness in improving physical and cognitive function, adherence, enjoyment, motivation, and mental health; promoting physical activity, digital literacy, and social engagement; identifying mild cognitive impairment; and reducing suicidal ideation.

Game elements used were ranked by frequency as progress, challenges, goals, levels, rewards, sensation, storytelling or narration, leaderboard, surprise, and avatar. However, due to insufficient information in these studies, it remains unclear whether intervention outcomes should be attributed to single game elements or synergistic effects of multiple elements. mHealth types included augmented and VR-based training systems, wearable devices, mobile phones, tablets, and Windows platforms and devices. Notably, only 4 studies applied theoretical frameworks (behavioral economic principles and gamification theory, technology acceptance model, and human-centered design framework), and 3 omitted the concrete approach to gamification. Given these findings, there is substantial potential for wider adoption of mHealth-based gamification interventions for clinicians and patients in future health promotion among older adults.

### Intervention Characteristics

First, all included studies in this research had relatively short intervention periods, with the longest lasting only 14 weeks [39], and 1 study even involved a single intervention lasting just 5 to 10 minutes [44]. This finding aligns with Aschentrup et al [31], where only 2 studies had a 12-week intervention period. In reality, the long-term effects of interventions are one of the most critical aspects for evaluating intervention efficacy. Additionally, most studies did not report specific details of intervention dosage, particularly intervention frequency and the duration of single sessions. Therefore, future research should explore long-term effects and report intervention dosage details to facilitate study reproducibility.

Second, the mHealth types involved in the studies included augmented and VR-based training systems, wearable devices, mobile phones, tablets, and Windows platforms and devices, with mobile phones and tablets being the most commonly used. This contradicts the findings of Xu et al [49], whose study indicated that most research used wearable devices. A possible reason for this discrepancy is that their study focused solely on physical activity in participants, and wearable devices can provide real-time feedback on daily steps and energy expenditure through specially designed algorithms [50,51]. Thus, when using mHealth-based gamification interventions to promote physical activity among older adults in the future, we recommend prioritizing wearable devices as the mHealth type.

Finally, in terms of theoretical applications, only 4 studies applied theoretical frameworks: 1 used behavioral economic principles to select gamification elements [40], 1 used gamification theory to guide intervention design [45], 1 used the technology acceptance model to explore older adults’ experiences with the gamified mobile app, and 1 adopted a human-centered design framework to guide the development of an mHealth-based gamified intervention system. This finding differs from previous research. For example, Montagni et al [52] noted that almost all included studies were grounded in robust theories, which were proven effective in promoting understanding and evaluating information related to vaccines and behavioral change end points. Literature reviews also show that theory-driven interventions are more effective than non–theory-based ones [53]. Therefore, we advocate that future designs of mHealth-based gamification interventions for older people’s health promotion should be closely integrated with theoretical frameworks.

### Gamification Characteristics

First, in this study, the most frequently used game element was progress, followed by challenges and goals. The most commonly used game elements align with a previous study [49]. Notably, goals serve as a key technique for behavioral change [54]. When combined with progress and feedback, they can significantly boost intrinsic motivation [55]. The only game element unused in all included studies was social sharing, which contradicts prior findings. For example, Xu et al [49] showed that when using mHealth-based gamification interventions for physical activity, the second most common game element was social interaction, which not only enhanced users’ enjoyment but also promoted motivation for physical activity through social incentives. The discrepancy might be attributed to differing inclusion criteria for gamification elements across studies [56,57]. Additionally, Mahmoudi et al [38] emphasized the importance of considering user preferences and personalized game elements, which was not reflected in our study. Thus, developers and researchers are encouraged to integrate social interaction, user preferences, and personalized gamification elements into interventions to enhance user engagement and, in turn, their effectiveness in health-related outcomes.

Second, gamification is one of the most critical components of mHealth-based gamification interventions. However, it is worth noting that among the 11 included studies, 3 failed to describe how gamification was specifically implemented in mHealth. This could undermine the reproducibility and comparability of research findings, thereby hindering the accurate evaluation of intervention efficacy and the formulation of evidence-based guidelines. Such oversight also aligns with previous review studies [31,38,58]. Thus, future research is recommended to establish corresponding reporting guidelines, emphasizing the need to strengthen this aspect of reporting for field advancement.

Finally, it is noteworthy that each study explained the reasons for using game elements, primarily, including promoting engagement and motivation, enhancing adherence, increasing enjoyment, and improving intervention efficacy. These findings are consistent with previous studies. For example, Mahmoudi et al [38] noted that most studies included in their research aimed to use game elements to enhance the adherence and motivation of children with disabilities. Additionally, a study has found that introducing gamified applications can improve intervention effectiveness by encouraging users to use them continuously and frequently [59].

### Needs and Experiences

Only 3 studies collected older users’ needs and experiences. The positive feedback can be categorized into 4 main aspects: first, the attributes of interventions, covering practical design aspects such as user-friendly design, personalization, and accessibility for play at any time; second, user engagement and subjective experience, which included high acceptability, satisfaction, emotional satisfaction, enhanced enjoyment, motivation to continue use, and a refreshing experience; third, health and functional benefits, such as perceived cognitive benefits and improvements in physical activity, memory, and sleep quality; and fourth, social and cultural elements, where the interventions’ social and collaborative aspects were described as valuable for fostering a sense of community. Possible explanations include that game elements enhance enjoyment and self-efficacy among participants. Additionally, integrating immersive and gamified elements provides dual benefits of cognitive and emotional stimulation, enhancing the overall user experience [2]. These results are also supported by previous studies [60,61].

Key issues included usability and technical flaws, including system freezes, difficult game mechanics, small character and object sizes, and movement misalignment, alongside reports of stress and initial physical fatigue. Usability is a critical aspect of improving acceptability and intervention adherence [48]. A prior study has shown that user-friendly interfaces, in particular, are a key factor in promoting positive acceptance among older users unfamiliar with new technologies [62]. Thus, future research should prioritize investigating needs and experiences, as this is crucial for designing mHealth-based gamification interventions. And more attention should be paid to user-centered design principles to ensure high acceptability and usability.

### Health Promotion Domains and Recommendations

Our study demonstrates that mHealth-based gamification interventions effectively improve physical and cognitive function, enhance adherence, promote physical activity, identify mild cognitive impairment, promote digital literacy and social engagement, improve mental health, reduce suicidal ideation, and enhance enjoyment and motivation among older adults.

These findings are consistent with previous studies. A study by Luo et al [63] showed that mHealth-based gamification interventions effectively improve pre-exposure prophylaxis adherence among men who have sex with men. Xu et al [49] found that mHealth-based gamification interventions may increase participation in physical activity. Huang et al [64] noted that using gamification to promote self-management among patients with chronic diseases has been widely recognized, including promoting physical exercise and rehabilitation training, enhancing motivation for symptom management, providing psychological support, improving cognitive function, and improving medication adherence. Additionally, research has indicated that when gamification is combined with emerging technologies, the findings may be more promising [65]. This could be attributed to digital tools being unconstrained by time and space [66], and gamification elements potentially enhancing their appeal and acceptability. Thus, future intervention designs should consider the integration of new technologies and gamification.

**Figure 3.**
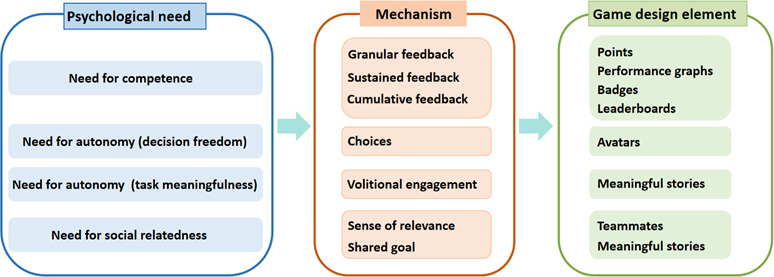
Psychological needs with matching game design elements.

The motivational effects of game elements can also be explained theoretically. Sailer et al [67] constructed a relationship between various gamification elements (such as points, leaderboards, and badges) and Ryan and Deci’s self-determination theory [68] (Figure 3). It is a theory of motivation that identifies 3 universal basic psychological needs that determine human behavior: competence, autonomy, and social inclusion [68]. If 1 or more of these needs are met (eg, through gamification elements), there is a positive impact on human behavior and the factors that influence it [67].

Beyond the topics identified in our review, prior research has demonstrated the potential of mHealth-based gamification interventions across other health domains. For example, Montagni et al [52] showed that digital gamification enhances vaccination knowledge and ultimately expands vaccination coverage. Another study highlighted its potential in promoting positive oral health behaviors [69]. To guide this future work, key methodological findings should also be considered. Xu et al [49] found that combining multiple game elements in mHealth-based gamification interventions is more effective than single elements, and theory-guided interventions outperform those without theoretical grounding. Therefore, we recommend that future empirical research should not only explore broader health domains but also (1) systematically compare the effects of single versus combined game elements and (2) ensure that the design and implementation of interventions are grounded in established theoretical frameworks.

### Practical and Research Implications

Our study suggests that mHealth-based gamification interventions are a promising approach for enhancing older adults’ health. Although this review identifies potential benefits, given the limited number of included studies, these findings should be interpreted with a cautious and critical perspective. We propose several recommendations for future research and practice:

First, to expand the innovative capacity outlined above, determining long-term impacts is crucial. Future studies should standardize research settings, increase sample sizes, extend intervention and follow-up periods, and conduct high-quality RCTs. They should also report intervention dosage details to facilitate study reproducibility.

Additionally, most existing studies lack theoretical frameworks. Thus, we advocate for integrating theoretical frameworks into future designs of mHealth-based gamification interventions for older health promotion, as theory-guided interventions may be more effective than nontheoretical ones.

Furthermore, future research is needed to explore whether intervention outcomes can be attributed to single game elements or synergistic effects of multiple elements.

Moreover, future research should establish corresponding reporting guidelines, particularly focusing on how gamification is specifically implemented in mHealth, thereby advancing the field and enhancing the reproducibility of studies.

Additionally, developers and researchers are encouraged to integrate social interaction, user preferences, and personalized gamification elements to create tailored, older-friendly interventions, thereby enhancing user engagement and health-related effectiveness.

Subsequently, future studies should prioritize investigating users’ needs and experiences, which are critical factors in designing mHealth-based gamification interventions.

Finally, empirical research for older adults should also expand mHealth-based interventions to broader health domains, such as exploring their impacts on the mental health of older populations.

### Limitations

We acknowledge several limitations in this review. First and foremost is the limited number of included studies, many of which were pilot studies or used small sample sizes. Consequently, the current evidence primarily supports the feasibility and preliminary potential of mHealth-based gamification rather than definitive broad effectiveness. Although we searched 8 databases to ensure comprehensiveness, the application of gamification in geriatric health remains a relatively emerging field.

Second, a major limitation of this scoping review is the overreliance on published peer-reviewed research, which may introduce publication bias and limit the comprehensiveness of the mapping. Relying solely on published works explicitly excludes gray literature such as technical reports, institutional documents, theses, dissertations, and government publications. These materials often provide valuable insights into early-stage developments and practical implementations. Although these contents may not have entered the formal academic literature, their contribution to this field is crucial. The absence of these perspectives may lead to an incomplete understanding of the overall landscape and real-world applications. While the focus of this review was deliberately on published and accessible peer-reviewed empirical evidence to ensure a high baseline of methodological rigor, future reviews should incorporate gray literature to capture a broader range of evidence, particularly for this emerging field.

Third, the protocol for this scoping review was not prospectively registered, which may diminish transparency and increase the potential for unintentional bias in our review process. To mitigate this, all authors critically reflected on the different steps. Fourth, restricting the search to English-language literature may have excluded relevant studies from non–English-speaking countries, potentially affecting the comprehensiveness and generalizability of our findings. Fifth, this review focused on describing effects and did not conduct a meta-analysis. Sixth, all included studies were conducted in high- or middle-income countries, limiting the generalizability of findings to low-income contexts. Furthermore, restrictions due to the specific definition of gamification adopted (ie, excluding serious games) may have limited the scope of relevant interventions included in this review. Finally, given the rapidly evolving digital landscape, the temporal limitation of the search period necessitates cautious interpretation as new research continues to emerge.

### Conclusion

As the first review to systematically scope this specific area, our study identifies mHealth-based gamification as a novel and viable strategy for fostering healthy aging. Although the findings are preliminary and require cautious interpretation due to the limited number of studies, the synthesized evidence highlights the capacity of these interventions to enhance enjoyment, motivation, cognitive function, physical activity, digital literacy, and other aspects among older adults. By bridging the gap between geriatric health needs and emerging digital technologies, this review provides a much-needed baseline for the field. However, to transform these emerging interventions into scalable real-world solutions, future research must prioritize rigorous, theory-driven, and long-term evaluations, alongside standardized reporting and personalized, socially interactive designs. Ultimately, this work lays the groundwork for developing more effective, age-tailored digital health tools that can tangibly improve the quality of life for the aging population.

## Supplementary material

10.2196/82368 Multimedia Appendix 1 Complete database search strategies, summary of intervention characteristics, and overview of gamification characteristics of included studies.

10.2196/82368 Multimedia Appendix 2 Frequency distribution of game elements across the 11 included studies.

10.2196/82368 Checklist 1 PRISMA-ScR checklist.

10.2196/82368 Checklist 2 PRISMA-S checklist.

## Abbreviations

MeSH: Medical Subject Headings
mHealth: mobile health
PRISMA: Preferred Reporting Items for Systematic Reviews and Meta-Analyses
PRISMA-S: Preferred Reporting Items for Systematic Reviews and Meta-Analyses Literature Search Extension
PRISMA-ScR: Preferred Reporting Items for Systematic Reviews and Meta-Analyses Extension for Scoping Reviews
RCT: randomized controlled trial
VR: virtual reality
