# Supplementary material for: mHealth-Based Gamification Interventions to Promote Health Among Older Adults: Scoping Review
Source: JMIR Mhealth Uhealth. 2026 May 4;14:e82368. doi: 10.2196/82368 (PMC13138710; doi:10.2196/82368)
Supplement: Multimedia Appendix 1 [file mhealth-v14-e82368-s001.docx]

**Appendix 1 Search syntax**

We employed for each database full and truncated search terms in the following search string:

**MHealth:** (telemedicine OR telehealth OR e-health OR m-Health OR eHealth OR mHealth OR "mobile applications" OR applications OR application OR app OR apps OR online OR mobile OR internet OR "web based" OR Smartphone OR "phone, smart" OR "smart phones" OR smartphones OR "smart phone" OR "phones, smart" OR "cell phone" OR iphone OR android OR iOS OR "Wearable Electronic Devices" OR website OR digital* OR system* OR electronic* OR technolog* OR device OR framework* OR "social support" OR Facebook OR "networks, social") AND

**Gamification**: (Gamification OR "game-based learning" OR gaming OR gamif* OR "game element*" OR game* OR "game mechanic*" OR gameful* OR "game design element*" OR "game-design element*" OR "game interface element*" OR "game feature" OR "game-like element*" OR "videogame element*") AND

**Older adults**: ("older adult*" OR "older people" OR elderly OR "older population" OR "senior citizen*")

Although the search strategy was the same for each database, suitable changes were made to accommodate for the different interfaces.

**Database 1: Pubmed**

Date searched: 10/12/2025

1.telemedicine[Mesh] OR telehealth[Title/Abstract] OR e-health[Title/Abstract] OR m-Health[Title/Abstract] OR eHealth[Title/Abstract] OR mHealth[Title/Abstract] OR "mobile applications" [Mesh] OR applications[Title/Abstract] OR application[Title/Abstract] OR app[Title/Abstract] OR apps[Title/Abstract] OR online[Title/Abstract] OR mobile[Title/Abstract] OR internet[Title/Abstract] OR "web based" [Title/Abstract] OR Smartphone[Mesh] OR "phone, smart" [Title/Abstract] OR "smart phones" [Title/Abstract] OR smartphones[Title/Abstract] OR "smart phone" [Title/Abstract] OR "phones, smart" [Title/Abstract] OR "cell phone" [Title/Abstract] OR iphone[Title/Abstract] OR android[Title/Abstract] OR iOS[Title/Abstract] OR "Wearable Electronic Devices" [Mesh] OR website[Title/Abstract] OR digital*[Title/Abstract] OR system*[Title/Abstract] OR electronic*[Title/Abstract] OR technolog*[Title/Abstract] OR device[Title/Abstract] OR framework*[Title/Abstract] OR "social support" [Mesh:noexp] OR Facebook[Title/Abstract] OR "networks, social" [Title/Abstract]

2.Gamification[Mesh] OR "game-based learning"[Title/Abstract] OR gaming[Title/Abstract] OR gamif*[Title/Abstract] OR "game element*"[Title/Abstract] OR game*[Title/Abstract] OR "game mechanic*"[Title/Abstract] OR gameful*[Title/Abstract] OR "game design element*"[Title/Abstract] OR "game-design element*"[Title/Abstract] OR "game interface element*"[Title/Abstract] OR "game feature"[Title/Abstract] OR "game-like element*"[Title/Abstract] OR "videogame element*"[Title/Abstract]

3. "older adult*"[Title/Abstract] OR "older people"[Title/Abstract] OR elderly [Title/Abstract] OR "older population"[Title/Abstract] OR "senior citizen*"[Title/Abstract]

4. #1 AND #2 AND #3

Filters applied: English, Humans, Aged: 65+ years, Exclude preprints.

**Results: 540**

**Database 2: Scopus**

Date searched: 10/12/2025

1.TITLE-ABS-KEY ( telemedicine OR telehealth OR e-health OR m-health OR ehealth OR mhealth OR "mobile applications" OR applications OR application OR app OR apps OR online OR mobile OR internet OR "web based" OR smartphone OR "phone, smart" OR "smart phones" OR smartphones OR "smart phone" OR "phones, smart" OR "cell phone" OR iphone OR android OR ios OR "Wearable Electronic Devices" OR website OR digital* OR system* OR electronic* OR technolog* OR device OR framework* OR "social support" OR facebook OR "networks, social" )

2.TITLE-ABS-KEY (gamification OR "game-based learning" OR gaming OR gamif* OR "game element*" OR game* OR "game mechanic*" OR gameful* OR "game design element*" OR "game-design element*" OR "game interface element*" OR "game feature" OR "game-like element*" OR "videogame element*" )

3.TITLE-ABS-KEY ("older adult*" OR "older people" OR elderly OR "older population" OR "senior citizen*" )

4. #1 AND #2 AND #3

Filters: English, Humans, Journal, Article or Review or Short survey

**Results: 1164**

**Database 3: Web of Science**

Date searched: 10/12/2025

1. AB=(telemedicine OR telehealth OR e-health OR m-Health OR eHealth OR mHealth OR "mobile applications" OR applications OR application OR app OR apps OR online OR mobile OR internet OR "web based" OR Smartphone OR "phone, smart" OR "smart phones" OR smartphones OR "smart phone" OR "phones, smart" OR "cell phone" OR iphone OR android OR iOS OR "Wearable Electronic Devices" OR website OR digital* OR system* OR electronic* OR technolog* OR device OR framework* OR "social support" OR Facebook OR "networks, social")

2. AB=(Gamification OR "game-based learning" OR gaming OR gamif* OR "game element*" OR game* OR "game mechanic*" OR gameful* OR "game design element*" OR "game-design element*" OR "game interface element*" OR "game feature" OR "game-like element*" OR "videogame element*")

3. AB=("older adult*" OR "older people" OR elderly OR "older population" OR "senior citizen*"))NOT (SILOID==("PPRN")

4. #1 AND #2 AND #3

Filters: English

**Results: 1731**

**Database 4: Embase**

Date searched: 10/12/2025

1. 'telemedicine'/exp OR 'telemedicine' OR 'telehealth'/exp OR 'telehealth' OR 'mobile application'/exp OR 'mobile application' OR 'internet'/exp OR 'internet' OR 'smartphone'/exp OR 'smartphone' OR 'social support'/exp OR 'social support'
2. 'e health':ti,ab,kw OR 'm health':ti,ab,kw OR ehealth:ti,ab,kw OR mhealth:ti,ab,kw OR 'mobile applications':ti,ab,kw OR applications:ti,ab,kw OR application:ti,ab,kw OR app:ti,ab,kw OR apps:ti,ab,kw OR online:ti,ab,kw OR mobile:ti,ab,kw OR 'web based':ti,ab,kw OR smartphone:ti,ab,kw OR 'phone, smart':ti,ab,kw OR 'smart phones':ti,ab,kw OR smartphones:ti,ab,kw OR 'smart phone':ti,ab,kw OR 'phones, smart':ti,ab,kw OR 'cell phone':ti,ab,kw OR iphone:ti,ab,kw OR android:ti,ab,kw OR ios:ti,ab,kw OR 'wearable electronic devices':ti,ab,kw OR website:ti,ab,kw OR digital*:ti,ab,kw OR system*:ti,ab,kw OR electronic*:ti,ab,kw OR technolog*:ti,ab,kw OR device:ti,ab,kw OR framework*:ti,ab,kw OR facebook:ti,ab,kw OR 'networks, social':ti,ab,kw
3. #1 OR #2
4. 'gamification'/exp OR 'game-based learning'/exp
5. gaming:ti,ab,kw OR gamif*:ti,ab,kw OR 'game element*':ti,ab,kw OR game*:ti,ab,kw OR 'game mechanic*':ti,ab,kw OR gameful*:ti,ab,kw OR 'game design element*':ti,ab,kw OR 'game-design element*':ti,ab,kw OR 'game interface element*':ti,ab,kw OR 'game feature':ti,ab,kw OR 'game-like element*':ti,ab,kw OR 'videogame element*':ti,ab,kw
6. #4 OR #5
7. 'older adult*':ti,ab,kw OR 'older people':ti,ab,kw OR elderly:ti,ab,kw OR 'older population':ti,ab,kw OR 'senior citizen*':ti,ab,kw
8. #3 AND #6 AND #7
9. #3 AND #6 AND #7 AND [english]/lim AND ([aged]/lim OR [very elderly]/lim) AND [humans]/lim AND [embase]/lim

**Results:44**

**Database 5: Cochrane Library**

Date searched: 10/12/2025

#1 MeSH descriptor: [Telemedicine] explode all trees

#2 MeSH descriptor: [Mobile Applications] explode all trees

#3 MeSH descriptor: [Internet] explode all trees

#4 MeSH descriptor: [Smartphone] explode all trees

#5 MeSH descriptor: [Cell Phone] explode all trees

#6 MeSH descriptor: [Wearable Electronic Devices] explode all trees

#7 MeSH descriptor: [Social Support] explode all trees

#8 #1 OR #2 OR #3 OR #4 OR #5 OR #6 OR #7

#9 (elehealth OR e-health OR m-Health OR eHealth OR mHealth OR applications OR application OR app OR apps OR online OR mobile OR internet OR "web based" OR "phone, smart" OR "smart phones" OR smartphones OR "smart phone" OR "phones, smart" OR iphone OR android OR iOS OR website OR digital* OR system* OR electronic* OR technolog* OR device OR framework* OR Facebook OR "networks, social"):ti,ab,kw

#10 #8 OR #9

#11 MeSH descriptor: [Gamification] explode all trees

#12 ("game-based learning" OR gaming OR gamif* OR "game element*" OR game* OR "game mechanic*" OR gameful* OR "game design element*" OR "game-design element*" OR "game interface element*" OR "game feature" OR "game-like element*" OR "videogame element*"):ti,ab,kw

#13 #11 OR #12

#14 ("older adult" OR "older people" OR elderly OR "older population" OR "senior citizen"):ti,ab,kw

#15 #10 AND #13 AND #14

Filters: English

**Results: 301**

**Database 6: CINAHL**

Host: EBSCOhost

Date searched: 10/12/2025

1.AB (telemedicine OR telehealth OR e-health OR m-Health OR eHealth OR mHealth OR "mobile applications" OR applications OR application OR app OR apps OR online OR mobile OR internet OR "web based" OR Smartphone OR "phone, smart" OR "smart phones" OR smartphones OR "smart phone" OR "phones, smart" OR "cell phone" OR iphone OR android OR iOS OR "Wearable Electronic Devices" OR website OR digital* OR system* OR electronic* OR technolog* OR device OR framework* OR "social support" OR Facebook OR "networks, social")

2.AB (gamification OR game OR "game-based learning" OR gamif* OR "game element*" OR game* OR "game mechanic*" OR gameful* OR "game design element*" OR "game interface element*" OR "game feature" OR "game-like element*" OR "videogame element*")

3. AB ("older adult" OR "older people" OR elderly OR "older population" OR "senior citizen")

4. #1 AND #2 AND #3

Filters: English, aged: 65+ years, Academic (peer-reviewed) journals

**Results: 254**

**Database 7: PsycARTICLES**

Date searched: 10/12/2025

1. (telemedicine OR telehealth OR e-health OR m-Health OR eHealth OR mHealth OR "mobile applications" OR applications OR application OR app OR apps OR online OR mobile OR internet OR "web based" OR Smartphone OR "phone, smart" OR "smart phones" OR smartphones OR "smart phone" OR "phones, smart" OR "cell phone" OR iphone OR android OR iOS OR "Wearable Electronic Devices" OR website OR digital* OR system* OR electronic* OR technolog* OR device OR framework* OR "social support" OR Facebook OR "networks, social")

2. (Gamification OR "game-based learning" OR gaming OR gamif* OR "game element*" OR game* OR "game mechanic*" OR gameful* OR "game design element*" OR "game-design element*" OR "game interface element*" OR "game feature" OR "game-like element*" OR "videogame element*")

3. ("older adult*" OR "older people" OR elderly OR "older population" OR "senior citizen*")

4. #1 AND #2 AND #3

Filters: English, aged: 65+ years, Academic (peer-reviewed) journals

**Results:40**

**Database 8: IEEE Xplore**

Date searched: 10/12/2025

1. "Abstract":telemedicine OR "Abstract":telehealth OR "Abstract":e-health OR "Abstract":m-Health OR "Abstract":eHealth OR "Abstract":mHealth OR "Abstract":"mobile applications" OR "Abstract":applications OR "Abstract":application OR "Abstract":app OR "Abstract":apps OR "Abstract":online OR "Abstract":mobile OR "Abstract":internet OR "Abstract":"web based" OR "Abstract":Smartphone OR "Abstract":"phone, smart" OR "Abstract":"smart phones" OR "Abstract":smartphones OR "Abstract":"smart phone" OR "Abstract":"phones, smart" OR "Abstract":"cell phone" OR "Abstract":iphone OR "Abstract":android OR "Abstract":iOS OR "Abstract":"Wearable Electronic Devices" OR "Abstract":website OR "Abstract":digital* OR "Abstract":system* OR "Abstract":electronic* OR "Abstract":technolog* OR "Abstract":device OR "Abstract":framework* OR "Abstract":"social support" OR "Abstract":Facebook OR "Abstract":"networks, social"

2."Abstract":gamification OR "Abstract":game OR "Abstract":"game-based learning" OR "Abstract":gamif* OR "Abstract":"game element*" OR "Abstract":game* OR "Abstract":"game mechanic*" OR "Abstract":gameful* OR "Abstract":"game design element*" OR "Abstract":"game interface element*" OR "Abstract":"game feature" OR "Abstract":"game-like element*" OR "Abstract":"videogame element*"

3. ("Abstract":"older adult*" OR "Abstract":"older people" OR "Abstract":elderly OR "Abstract":"older population" OR "Abstract":"senior citizen*")

4. #1 AND #2 AND #3

Filters Applied: Journals

**Results: 38**

**Appendix 2 Tables**

**Table S1. Summary of intervention characteristics of the included studies.**

| Author (year) | Country | Study Design | Study Objectives | Participant Characteristics | | | Intervention Characteristics | | | | |
| --- | --- | --- | --- | --- | --- | --- | --- | --- | --- | --- | --- |
|  |  |  |  | Mean (±SD)  Age | Percentage of Female Participants(%) | Target  Population | Sample  Size | Study  Setting | Type of  Mhealth Used | Theory  Used | Duration |
| Lee, Eun-Lee et al  (2024) | Korea | RCT | To confirm the safety and efficacy of information technology convergence gamification exercise equipment | 71.20±4.31 | 60.00 | Community-Dwelling  Older People | 40 | Community | Augmented reality-based training system | NS | 8 weeks, 3 times a week, 30 minutes a time |
| Greysen, S. Ryan et al  (2024) | USA | RCT | In this study, we tested a behaviorally designed gamification intervention with adults at risk for AD/ADRD based on their age and genetic risk factors. | 70.40±3.00 | 78.00 | Adults at risk for Alzheimer’s  disease and Alzheimer’s disease and related dementias | 94 | NS | ①Wearable device  ②Mobile phone (application) | Behavioral economic principles | 12-week |
| Roh, Hyun Woong et al  (2022) | Korea | RCT | To test the effectiveness of an eight-week smartphone application-based motivation enhancement program for inducing brain health behavior. | Mean age: 73.30 years  (range= 66.80–79.80) | 65.00 | NS | 49 | Hospital | Mobile phone (application) | Gamification  theory | 8-week |
| Murata, Naohiro et al (2025) | Japan | Quasi-Experiment | To evaluate a gamified mobile application for the early detection of cognitive impairment associated with dementia. | 75.80±10.80 | 69.00 | Inpatients | 138 | Hospital | Mobile phone (application) | NS | Each solo  gameplay session lasted approximately  5-10 min |
| Aniket Nagle, Robert Riener et al  (2015) | Switzerland | Quasi-Experiment | To investigate the overall effect of a specific joint set of game element implementations. | 69.90 ± 6.40 | 47.62 | Autonomously living older adults | 21 | Home | Tablet | NS | 3 weeks |
| Kamnardsiri, Teerawat et al  (2024) | Thailand | Mixed study | To develop and evaluate the usability of a low-intensity, gamification-based, interactive physical-cognitive exercise for older adults in a home-based setting. | 65.27 ±4.40 | 53.33 | Community-dwelling  older adults | 15 | Home | Windows platform and  devices（the notebook or personal computer） | NS | 60 minutes per day,  3 days per week,  for 4 weeks |
| ChePa, Noraziah, et al (2022) | Malaysia | Quasi-Experiment | This article focuses on how game can  be utilized in providing psychotherapy intervention to elderly with memory disorders issues. | 66.40±5.62 | 60.00 | Showing early symptoms  of memory disorder | 15 | The care-center | Tablet | NS | 14 weeks |
| Nacimiento‑García, Eduardo, et al （2024） | Spain | Mixed study | This study focuses on developing and implementing a practical and tailored gamified platform to foster active aging in older adults. | 64.17±5.51 | 91.67 | NS | 12 | University | Tablets and Mobile phone (application) | NS | NS |
| Lai, Frank Ho-Yin, et al  (2025) | China | Qualitative study | This study explores the acceptability and feasibility of a Cantonese-language gamified mobile application, Challenges intown, designed to support cognitive training through real-life simulations. | 76.23±8.67 | 55.00 | Older adults with basic familiarity with mobile devices and no diagnosed cognitive impairments | 20 | Community | Mobile phone (application) | Technology acceptance model | NS |
| Chien, Shih-Ying et al (2025) | China | Quasi-Experiment | This study aimed to investigate the feasibility and usability of a novel home-based PTR system (Intelligent Pulmonary Rehabilitation Exercise System (IPRES)), eliciting user experience reported by a cohort of outpatients with relevant respiratory diseases. | 65.70±6.40 | 58.33 | High-risk, post-discharge CPD patients, predominantly GOLD stage 3 | 36 | Hospital | ①Tablet  ②Wearable device | Human-centered design framework | NS |
| Zhang, Jinjin (2025) | China | RCT | This study aimed to examine the effectiveness of gamified group-based physical activity in a virtual reality (VR) environment for enhancing psychological resilience and reducing suicidal ideation among elderly women. | The participants were all aged 60 and above, but the average age is NS. | 100.00 | Elderly women experiencing suicidal ideation | 120 | Community | Virtual reality based training system | NS | 5-week, 3 times per week, 40-60 min a time. |

RCT: Randomized Controlled Trial; NS: Not Specified

**Table S2. Detailed overview of gamification characteristics in the included studies.**

| Author (year) | Definition of gamification | Gamification elements | Reasons for using gamification elements | The concrete approach to gamification |
| --- | --- | --- | --- | --- |
| Lee, Eun-Lee et al  (2024) | NS | ①Levels  ②Progress  ③Challenges | ①Increase enjoyment  ②Promote engagement and motivation  ②Enhance intervention sustainability | The difficulty level was sequentially changed from “easy” during the first week, to “normal” during the second and third weeks, to “difficult” during weeks 4-8. |
| Greysen, S. Ryan et al  (2024) | Gamification is the application of game design elements in non-game contexts. | ①Goals  ②Levels  ③Progress  ④Reward  ⑤Challenges | ①Facilitate sustained increases in physical activity | ①On days when participants met their step goal, they received a congratulatory message the following morning, which provides immediate gratification. On days that participants do not meet their step goals; they receive a message on the following morning that they lost 10 points from their balance. ②At the end of each week, participants could move up a level (from lowest to highest: blue, bronze, silver, gold, platinum) if they retained 40 points or move down a level if they did not. This design creates achievable goal gradients (the notion that the next highest level is attainable), a sense of status with accomplishment, and progression through the game. Fourth, participants started at the silver level so they could experience either the accomplishment of rising to gold or the loss of dropping to bronze upon completing the first week of the intervention. |
| Roh, Hyun Woong et al  (2022) | Gamification theory is a method that applies gaming mechanisms to non-gaming contexts. | ①Goals  ②Levels  ③Progress  ④Challenges ⑤Leaderboard  ⑥Storytelling/Narration | ①Positively impact health-related interventions ②Promote engagement and motivation | ① Achievement levels were categorized as gold and silver medals; participants selected either one during self-rating.  ②Participants could see their achievement in the leader board of the smartphone application. The leader board can stimulate participants to achieve a goal as last week, or to make an improvement than last week. |
| Murata, Naohiro et al (2025) | NS | ①Goals  ②Progress  ③Challenges  ④Reward  ⑤Storytelling/Narration | ①Help overcome distress in traditional cognitive tests  ②Enhance adherence  ③Enhance early detection rates | ①The game uses the N-back task, gradually increasing difficulty by requiring players to memorize N additional ingredients after correctly fulfilling N orders. In-game revenue reflects performance based on correct orders. |
| Aniket Nagle, Robert Riener et al  (2015) | NS | ①Goals  ②Levels  ③Progress  ④Reward  ⑤Challenges  ⑥Sensation  ⑦Storytelling/Narration | ①Promote engagement and motivation  ②Boost performance  ③Increase enjoyment | NS |
| Kamnardsiri, Teerawat et al  (2024) | Gamification,  which involves using game-based mechanics to motivate  individual action and learning for a specific target. | ①Goals  ②Levels  ③Progress  ④Challenges  ⑤Reward  ⑥Leaderboard  ⑦Sensation | Enhance adherence | NS |
| ChePa, Noraziah et al (2022) | NS | ①Goals  ②Progress  ③Reward  ④Sensation  ⑤Surprise  ⑥Storytelling/Narration | ①Top nonpharmacological therapy for memory disorders  ②Improve therapeutic effect of recovery exercises | NS |
| Nacimiento‑García, Eduardo et al (2024) | Gamification, rooted in applying game elements and principles  to non-gaming contexts. | ①Goals  ②Levels  ③Progress  ④Challenges  ⑤Reward  ⑥Surprise  ⑦Leaderboard  ⑧Sensation  ⑨Storytelling/Narration  ⑩Avatar | ①Provide a realistic and captivating user experience  ②Drive behavioral changes  ③Promote engagement and motivation | ①Scoring System：Users earn points by completing games. As users accumulate points, they advance through levels and unlock achievements, fostering continuous engagement.  ②Level Scaling：Each level has a specific point threshold that users must meet or exceed to advance. Levels are represented by unique badges displayed as avatars on users’ profiles, providing a visual indicator of progress and adding status within the platform.  ③Unlocking Achievements：Achievements are visually represented as badges on the user’s profile, offering additional motivation.  ④Ranking：The leaderboard displays user scores in descending order, highlighting the top performers.  ⑤Progressive Difficulty in Games：Each game and challenge on the platform were designed with a progressive difficulty structure  ⑥Immersive Challenges with Gradual Engagement：These challenges involve interactive 360◦ virtual tours of famous landmarks.  ⑦Continuous Feedback and Score Tracking：Immediate feedback after each interaction allows users to know whether their answers are correct. |
| Lai, Frank Ho-Yin, et al (2025) | NS | ①Reward  ②Sensation  ③Progress  ④Challenges  ⑤Level | ①To make cognitive training both enjoyable, effective and less intimidating.  ②Promote engagement and motivation | 1. It includes a level-based progression system that gradually increases task complexity, allowing users to build skills over time. 2. Reward mechanisms, the three-tier virtual trophy, are integrated to reinforce success and maintain motivation. 3. Users receive real-time feedback through visual and auditory cues, helping them understand their performance and encouraging learning through immediate reinforcement. 4. An adaptive difficulty system ensures that each user is met with the right level of challenge, promoting sustained engagement and neuroplasticity. 5. Tracks user performance and cognitive improvements. |
| Chien, Shih-Ying et al (2025) | NS | ①Challenges  ②Sensation  ③Progress | ①Promote engagement and motivation  ②Strengthen self-efficacy. | ① Tablet-based application (Android OS): Delivers gamified rehabilitation modules with interactive challenges and real-time visual feedback.  ② Gamification: Interactive challenges, scenic environments, and progress tracking foster engagement and enjoyment. |
| Zhang, Jinjin (2025) | Gamification, defined as the deliberate use of game design elements—including point systems, progressive levels, leaderboards, rewards, and instant feedback—within non-game settings, has been shown to enhance motivation and engagement. | ①Level  ②Sensation  ③Reward  ④Progress | ①Promote engagement and motivation  ②Maximize social connection  ③Enhance adherence  ④Increase enjoyment  ⑤Boost confidence and reinforce a sense of importance and achievement.  ⑥Mitigate isolation, loneliness, and worthlessness. | Each session featured gamification elements such as a ranking system, scoring mechanisms, and personalized incentives (e.g., visual rewards, nostalgic video clips, and public recognition of progress in front of family members). |

NS: Not Specified
