# Supplementary figures and images for: mHealth-Based Gamification Interventions to Promote Health Among Older Adults: Scoping Review
Source: JMIR Mhealth Uhealth. 2026 May 4;14:e82368. doi: 10.2196/82368 (PMC13138710; doi:10.2196/82368)

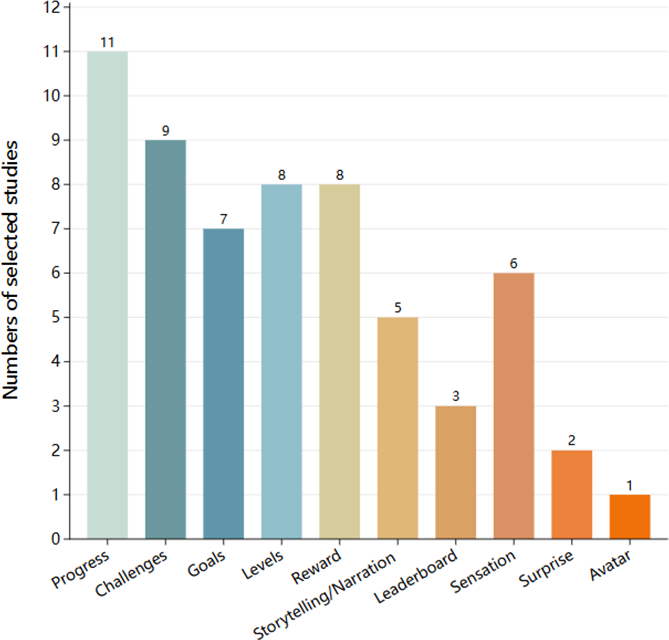

Supplement: Multimedia Appendix 2 [file mhealth-v14-e82368-s002.png]
